# Dosage of Sulfidized Nano Zero-Valent Iron, Soil Moisture and pH Influences on Fraction of Arsenic and Cadmium in Contaminated Paddy Soil

**DOI:** 10.3390/nano15231768

**Published:** 2025-11-25

**Authors:** Jiabing Wu, Jianxiong Xie, Hang Wei, Pengran Guo, Zhiliang Chen

**Affiliations:** 1 Chinese Research Academy of Environmental Sciences, Beijing 100012, China; 2 Guangdong Engineering Technology Research Center of Heavy Metal Pollution Control and Restoration in Farmland Soil, South China Institute of Environmental Sciences, Ministry of Ecology and Environment, Guangzhou 510535, China; 3 Guangdong Provincial Key Laboratory of Chemical Measurement and Emergency Test Technology, Institute of Analysis, Guangdong Academy of Sciences (China National Analytical Center, Guangzhou), Guangzhou 510070, China

**Keywords:** sulfurized nanoscale zero-valent iron, arsenic, cadmium, synchronous passivation, paddy soil

## Abstract

Rice (*Oryza sativa* L.) exhibits a heightened ability to bioaccumulate arsenic (As) and cadmium (Cd), which directly affects the quality of agricultural products and poses serious risks to both the ecological environment and human health. Due to considerable differences in the occurrence states and chemical behaviors of As and Cd, simultaneous remediation efforts for water or soil contaminated by these elements often prove challenging. Our previous study indicated that the addition of both As and Cd markedly promoted the immobilization of each other by sulfidized nano-zero-valent iron (S-nZVI). To further explore the influence of S-nZVI on the passivation of As-Cd composite contamination, we examined its effect on the residual proportions of As and Cd in the soil by varying the dosage of S-nZVI, the soil moisture content and pH levels. At 2 g·kg^−1^ S-nZVI over a 90-day period, residual fraction reached 83% for As and 39% for Cd. When the water content was 100%, residual fractions peaked at 83% for As and 29% for Cd. Additionally, variations in initial pH levels were found to have no significant impact on the remediation efficiency of As and Cd. This suggests that S-nZVI has the ability to sustain the stabilization of As and Cd in soil across diverse environmental conditions. The evident passivation effects on As-Cd composite contaminated soil can effectively reduce the potential ecological risk associated with these contaminants.

## 1. Introduction

The intensification of mining and metallurgical activities, accelerated industrialization, and rapid socio-economic growth in China have expanded industrial production. This expansion has led to widespread heavy metal contamination in Chinese soils [1,2]. Many studies have shown that environmental heavy metal contamination is predominantly attributable to anthropogenic activities, such as sewage irrigation, sludge application, mining and smelting of metal ores, and the application of phosphate fertilizers [3,4]. According to the national soil contamination survey released in 2014, about 20 million hectares of arable land in China are affected by different levels of heavy metal contamination. Among all sampling sites, 19.4% exceeded the national standards. Cadmium (Cd) and arsenic (As) were the main heavy metals exceeding the limits, with exceedance rates of 7.0% and 2.7%, respectively. The spatial distribution of As and Cd in Chinese soils varies among regions, but there is considerable overlap in some areas. The carcinogenic risk is higher in the southern region than in the northern region [1,5]. Soil samples collected from 19 provinces in four major rice-producing regions showed that 33.6% and 6.19% of soils exceeded the standard limits for Cd and As, respectively [6]. In the Pearl River Delta region, 15% of rice samples exceeded the Cd limit, and 22% exceeded the As limit [7]. Therefore, contamination of paddy soils with As and Cd has become a serious threat to the safety of agricultural products in China.

Rice is a major food crop and an important dietary source of inorganic arsenic (As) and cadmium (Cd), two toxic contaminants of global concern [8,9]. The intermittent flooding of paddy fields creates anaerobic conditions that significantly alter soil redox potential (Eh) and pH levels. These changes markedly influence the biogeochemical processes of several elements, such as As and Cd [10]. Rice grains can accumulate over 10 times more As than other cereals, posing serious risks to human health [11,12]. Because As and Cd exhibit distinct biogeochemical behaviors in paddy systems, it is difficult to develop unified strategies to simultaneously reduce their accumulation in rice seeds [10].

Nano-zero-valent iron (nZVI) has been effectively utilized as an adsorbent for heavy metal ion removal [13]. Its reaction rate constant for As immobilization can be up to 1000 times higher than that of normal zero-valent iron (ZVI) [14,15]. However, its ability to immobilize Cd is relatively poor and less stable [16]. Several factors limit the environmental performance of nZVI [17]. Due to its minute particle size and extensive specific surface area, nZVI tends to agglomerate under magnetic interactions [18,19]. In addition, its high reactivity promotes rapid oxidation and the formation of a passivating iron oxide layer, which reduces effective contact with contaminants [20]. Furthermore, hydrogen ions and dissolved oxygen may compete with pollutions for electrons, decreasing treatment efficiency [21]. Various modification methods have been developed to improve nZVI performance [22,23,24]. For example, graphene-loaded nano zero-valent iron (nZVI/rGOs) demonstrated enhanced Cd(II) immobilization capabilities (425.72 mg·g^−1^) [25]. Combining nZVI with oxidants such as persulfate (PS), peroxymonosulfate (PMS), or hydrogen peroxide (HP) can also improve As(III) removal (III) immobilization. During these reactions, Fe^0^ generates various reactive oxygen species (·OH, SO_4_^−^·), partially oxidizing As(III) to the less toxic As(V) [22]. Additionally, introducing other metallic materials into nZVI can enhance its reactivity and accelerate reduction processes [24]. Among these modification strategies, sulfurization stands out as a simple and environmentally friendly approach [26].

Studies have demonstrated that sulfur-modified nano zero-valent iron (S-nZVI) greatly enhances the immobilization of As and Cd in water compared to nZVI alone [27,28]. The Cd removal capacity of S-nZVI in aqueous solutions can reach up to 495 mg·g^−1^, nearly an order of magnitude higher than that of unmodified nZVI. Unlike the adsorption process dominated by iron oxides, Cd immobilization by S-nZVI mainly occurs through the formation of stable CdS compounds, where Cd^2+^ ions replace Fe in FeS structures within the nanoparticles [17]. Cd(II) can also bind to FeS or sulfhydryl groups (FeSH^+^), forming Fe_(1−x)_Cd_x_S compounds, with immobilization capacities up to 85 mg·g^−1^ [29]. The mechanism of As immobilization by S-nZVI is more complex. Adsorbed As onto S-nZVI can react with sulfur species to form sulfide precipitates [30], increasing immobilization efficiency from 60% to 98% [31]. As(III) is mainly immobilized via specific inner-sphere complexation on the surface through a process strongly influenced by pH levels [32]. Meanwhile, the primary component adsorbed on the surface of S-nZVI is As(V), which is because Fe^0^ in S-nZVI can react with molecular oxygen producing hydroxyl radicals (·OH), which oxidize As(III) to As(V) [26]. Overall, As(III) and Cd(II) immobilization involves combined mechanisms of surface adsorption, oxidation, and co-precipitation [29,30].

Our previous study indicated that S-nZVI exhibits a synergistic effect during the simultaneous immobilizations of As and Cd. Electrostatic adsorption and ternary complex formation were identified as the main mechanisms driving this synergy [33]. Simultaneous treatment further improved overall immobilization efficiency. In addition, pH changes directly influence the chemical states of heavy metal states in soil and affect their bioavailability [34,35]. Contaminated soil containing both As and Cd exhibit opposing changes in biological activity along varying pH and redox potential (Eh) levels [36]. Increasing pH decreases Cd mobility but enhances As mobility, whereas lowering pH has the reverse effect [37]. Different water management also modifies soil redox conditions and pH, thereby affecting the transport, transformation, and bioavailability of these contaminants [11,38,39]. However, studies focusing specifically on remediation strategies targeting composite contamination arising from As-Cd interactions utilizing S-nZVI remain limited. While previous studies have elucidated the immobilization mechanisms and efficiencies of S-nZVI for As and Cd primarily in aqueous systems or under single-factor control, its practical behavior and mechanistic responses in complex soil environments remain poorly understood. Existing research often isolates one variable at a time, which cannot fully capture the coupled effects of realistic field conditions. This study distinguishes itself by systematically quantifying how key field-manageable factors, S-nZVI dosage, soil moisture regime (as a proxy for redox potential) and pH, individually and interactively influence the fractionation, transformation, and bioavailability of As and Cd in co-contaminated paddy soils. The novelty of this work lies in providing mechanistic insights into how the coordinated adjustment of these parameters can optimize S-nZVI performance for the simultaneous stabilization of antagonistically behaving As and Cd. This integrated approach moves beyond confirming known S-nZVI behavior, offering a practical framework for tailoring amendment strategies under realistic paddy soil conditions.

Therefore, this study aimed to investigate practical applications surrounding the utilization efficacy of S-nZVI in soils co-contaminated with As and Cd. Specifically, it investigated how soil moisture content, S-nZVI dosage, and pH conditions influence the occurrence, chemical speciation, and mobility of As and Cd within paddy soils. In addition, changes in key soil physicochemical parameters such as Eh and pH were monitored to elucidate the mechanisms underlying S-nZVI-mediated stabilization. The findings are expected to provide new theoretical and practical guidance for optimizing S-nZVI-based remediation strategies in As-Cd co-contaminated agricultural soils.

## 2. Materials and Methods

All reagents used in this work were of analytical grade, and deionised water was employed as the solvent throughout the experiments. FeCl_3_·6H_2_O, Na_2_S·9H_2_O, CdCl_2_, HCl, NaOH, and NaAsO_2_ were sourced from Aladdin Reagents Co., Ltd. (Shanghai, China). KBH_4_ was obtained from Tianjin Kemiou Chemical Reagent Co., Ltd. (Tianjin, China). HNO_3_ and HClO_4_ were provided by Guangzhou Chemical Reagent Factory, while HF was supplied by Macklin Biochemical Co., Ltd. (Shanghai, China).

### 2.1. Collection, Preparation and Aging of Soil

The sample of As-contaminated soil was obtained from Zhaoqing, Guangdong Province. Large stones, roots and other impurities were removed prior to use. After drying in air, the soil passed through a 2 mm sieve and was then stored in a sealed container. The preparation of the Cd-contaminated soil consisted of adding CdCl_2_ solution (1.81 mg·kg^−1^) to the soil at a mass ratio of 1:1 (soil to water), followed by thorough mixing until the soil was completely air-dried. The dried soil was then pulverized into a fine powder and passed through a 2 mm diameter sieve. Finally, the soil was permitted to age naturally at room temperature for one month. The basic physicochemical properties of the test soil after aging are presented in Table 1.

### 2.2. Preparation of Sulfidized Nano Zero-Valent Iron

The S-nZVI was prepared via a one-step reduction process [28]. Initially, sodium borohydride solution (0.25 mol·L^−1^) was mixed with sodium sulfide, and then 500 mL of this mixture was gradually added to ferric chloride solution using a peristaltic pump operating at 45 rpm. This process occurred under continuous stirring with an electric stirrer set at 600 rpm. After all mixed liquid had been introduced, stirring continued for an additional 15 min to ensure a complete reaction. Subsequently, S-nZVI particles were collected using magnetic separation, washed twice with deionized water and once with anhydrous ethanol before being freeze-dried and stored under nitrogen gas at 4 °C. By adjusting the molar ratios of sulfur to iron in sodium sulfide (Na_2_S) and ferric chloride (FeCl_3_) solutions (0.045 mol·L^−1^), S-nZVI samples with varying S/Fe ratios can be produced. To eliminate dissolved oxygen from deionized water prior to use, nitrogen was bubbled through it for approximately 25 min.

### 2.3. Effect of S-nZVI Dosage on Remediation of As and Cd Contaminated Soil

Soil without any additive treatment served as the control group (CK). Different concentrations of S-nZVI, 1 g·kg^−1^, 2 g·kg^−1^, and 5 g·kg^−1^, were applied to soil, respectively. A total weight of 50 g of each soil sample underwent thorough mixing with corresponding amounts of S-nZVI before being placed into 100 mL centrifuge tubes containing deionized water, 100% of water-holding capacity (WHC); these tubes were sealed and incubated indoors away from light conditions at room temperature for further analysis after 0, 10, 30, 60 and 90 days. All experimental treatments were conducted in triplicates. Subsequently, the soil samples were collected, freeze-dried, ground and sieved for use.

### 2.4. Effect of Water Content on Remediation of As and Cd-Contaminated Soil

Using flooded soil as a control, the concentration of S-nZVI applied to the soil was 2 g·kg^−1^. A total of 50 g of soil was weighed and thoroughly mixed with S-nZVI. The soil was placed in 100 mL centrifuge tubes, and the soil moisture conditions were adjusted to 25%, 75% and 100% of the absolute moisture content; these tubes were sealed and incubated indoors away from light conditions at room temperature for further analysis after 0, 10, 30, 60 and 90 days. All experiments were conducted in triplicates. Subsequently, the soil samples were collected, freeze-dried, ground and sieved for use.

### 2.5. Effect of pH on Remediation of As and Cd Contaminated Soil

The initial pH of the soil was adjusted to 5, 5.6 and 8, respectively, using 0.1 mol/L HCl and 0.1 mol/L NaOH for pH adjustment. Meanwhile, the soil without pH adjustment was used as the control. The amount of S-nZVI added was 2 g·kg^−1^. The soil was weighed and thoroughly mixed with S-nZVI, loaded into 100 mL centrifuge tubes, and added with deionized water, 100% of water-holding capacity (WHC); these tubes were sealed and incubated indoors away from light conditions at room temperature for further analysis after 0, 10, 30, 60 and 90 days. All experiments were conducted in triplicates. Subsequently, the soil samples were collected, freeze-dried, ground and sieved for use.

### 2.6. Methods of Analysis

The soil pH was measured in a soil–water suspension of 1:2.5 (g soil/mL water) [40]. Fraction grading of soil As and Cd was classified by the BCR sequential extraction method, which extracts heavy metals in the weak acid soluble, reduced, oxidized and residual states through four steps [41]. The fractionation of As and Cd in soil was performed using the BCR sequential extraction procedure, which separates metals into four fractions: acid-soluble, reducible, oxidizable, and residual. All reagents were of analytical grade, including 0.11 mol/L acetic acid, 0.5 mol/L hydroxylamine hydrochloride (pH = 2.2), 30% H_2_O_2_, and 1 mol/L ammonium acetate (pH = 2). Briefly, 0.5 g of soil was extracted sequentially with the above reagents under constant-temperature shaking for 16 h at each step. After each extraction, the samples were centrifuged (1500× *g*, 5 min) and filtered through 0.45 μm membranes. The final residue was digested with mixed acids (HCl–HNO_3_–HF–HClO_4_) to determine residual metals. Concentrations are determined via Agilent 7800 ICP-MS systems (Agilent Technologies Co., Ltd., Santa Clara, CA, USA).

### 2.7. Statistical Analysis

All data were processed using Microsoft Excel 2021, presenting means and standard deviations, analyzed using IBM SPSS Statistics 27.0.1 (IBM Corp., Armonk, NY, USA) (LSD, *p* < 0.05) and plotted using Origin 2024 (OriginLab Corporation, Northampton, MA, USA).

## 3. Results and Discussion

### 3.1. Effects of S-nZVI Dosage on Soil As and Cd Fraction

**Figure 1:**
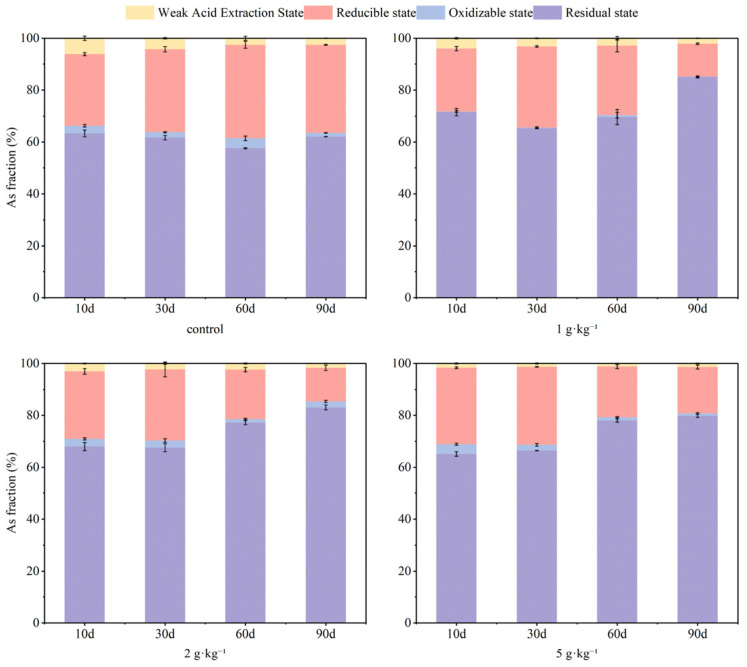
Changes in soil As forms with S-nZVI dosage before and after addition (total As concentration = 125.32 mg·kg^−1^; water content = 100%; S-nZVI concentration = 1 g·kg^−1^, 2 g·kg^−1^, 5 g·kg^−1^).

**Figure 2:**
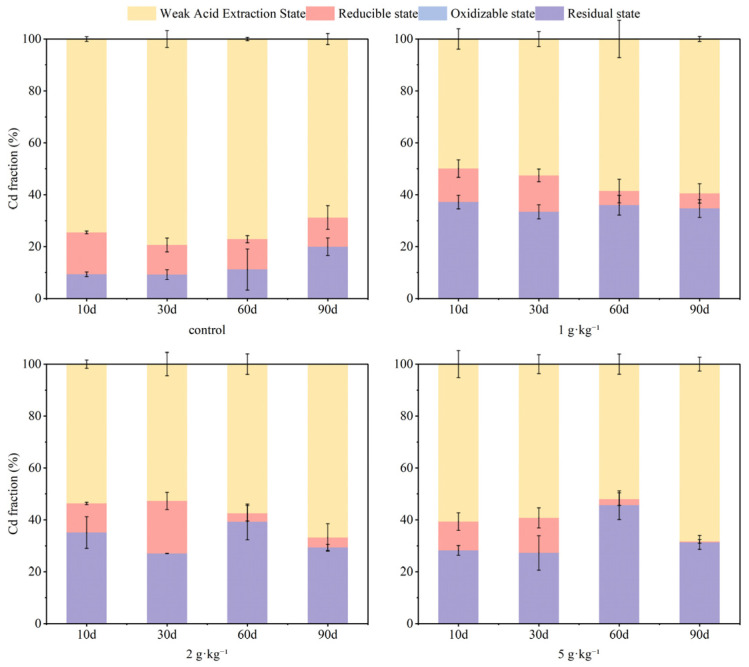
Changes in soil Cd forms with S-nZVI dosage before and after addition (total Cd concentration = 1.81 mg·kg^−1^; water content = 100%; S-nZVI concentration = 1 g·kg^−1^, 2 g·kg^−1^, 5 g·kg^−1^).

The incorporation of S-nZVI influenced the fraction of As and Cd, with variations in dosage impacting both the stability and remediation efficiency of soil As and Cd. Figure 1 illustrates the results from the BCR fraction grading of As in soil, revealing a decline in weak acid-extracted As content as S-nZVI dosage increased; specifically, a concentration of 5 g·kg^−1^ of S-nZVI effectively reduced weak acid-extracted As to less than 1% within the 90-day experimental period. Concurrently, this treatment facilitated the transformation of the most reduced state of As into poorly soluble Fe- and S-associated forms (e.g., FeAsO_4_, FeAsS, or As incorporated into Fe(III)-(oxyhydr)oxides) [32], accounting for up to 80% of the total As. S-nZVI possesses a distinctive core–shell structure composed of iron oxide and iron sulfide, which exhibits a more intricate mechanism for As immobilization. This mechanism includes surface adsorption and co-precipitation, wherein sulfide not only facilitates the precipitation process but also inhibits the formation of outer-layer complexes, thereby enhancing the efficiency of As immobilization [32]. Figure 2 presents findings from BCR fraction grading for Cd in soil. Given that S-nZVI surfaces are negatively charged in all soil pH environments, at the early stage of the experiment (0–30 days), S-nZVI exhibited superior immobilization capabilities for Cd compared to control groups. By day 60, an increase in residual Cd was observed alongside rising S-nZVI dosages, from 11% to 46%; however, by day 90, there was a notable transition from residual Cd to weakly acid-extracted forms following a clear dose-dependent trend. Due to its high solubility, the weak acid-extracted state of Cd is easily absorbed by plants and is regarded as a bioavailable form. A reduction in its content contributes to a decrease in soil Cd activity. In contrast, residual state Cd presents greater stability and poses challenges for plant absorption and utilization. The increase in its content indicates that Cd is further passivated [42]. In this study, applying S-nZVI into soil reduced weak acid extraction Cd and increased residue state concentrations, effectively achieving passivation and mitigating potential contamination risks associated with soil-bound Cd.

**Figure 3:**
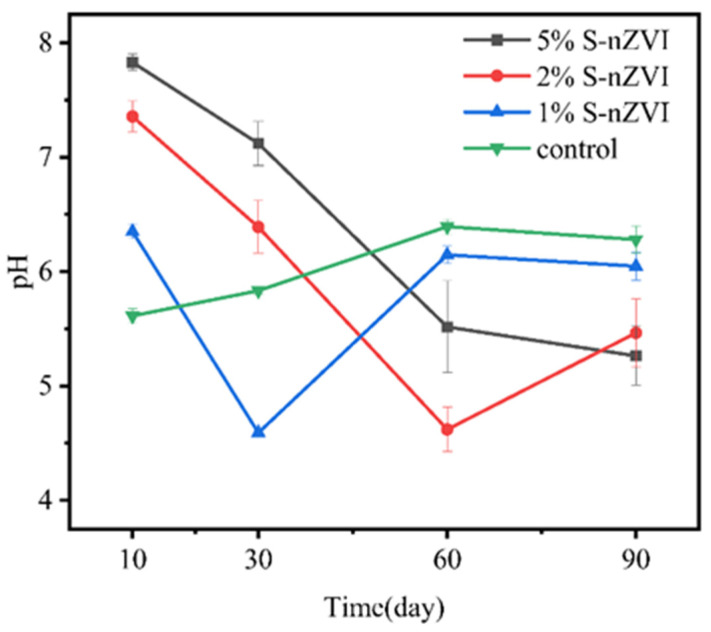
Changes in soil pH with S-nZVI dosage before and after addition (total As concentration = 125.32 mg·kg^−1^, total Cd concentration = 1.81 mg·kg^−1^, water content = 100%).

It is evident that the stabilization of As and Cd in soil to the residue state was enhanced with increasing dosage. This phenomenon can be partly attributed to the increased number of reactive sites resulting from higher S-nZVI dosages, which facilitated the adsorption of As and Cd onto the surfaces of Fe/Mn (hydr)oxides and sulfides, thereby achieving their stabilization [43]. The concentrations of As and Cd in the weak acid extractable state exhibited distinct trends across the low (1 g·kg^−1^), medium (2 g·kg^−1^), and high (5 g·kg^−1^) S-nZVI treatment groups, likely influenced by pH fluctuations throughout treatments. For As, as depicted in Figure 3, in the treatment group where S-nZVI was dosed at 1 g·kg^−1^, early additions resulted in elevated soil pH levels. This was attributed to the significant rise in ·OH content with increasing S-nZVI dosage and the simultaneous oxidation and reduction in As(III) within the nanoparticles [32]. Consequently, this change inhibited the stabilization of As, rendering the immobilization of soil As by S-nZVI less effective during the early stages of the experiment (0–30 days) compared to the control group due to electrostatic repulsion. Over time, as pH values decreased progressively, residues reflected increasing concentrations of stabilized As content, with a maximum of 85%. A similar situation was not observed within the higher dosage treatment group, suggesting more robust immobilization effects were achieved. In the experimental group containing higher concentrations of S-nZVI, the effect on Cd was the opposite. Specifically, the elevation seen initially due to increased pH fostered improved stabilization outcomes, whereas subsequent reductions prompted the reducible state of Cd in the soil to transform into other chemical forms and eventually dropped to 0 at 30–60 days. At 90 days, the residual concentration of Cd was reduced. The influence of S-nZVI on Cd was particularly evident in the experimental group with a higher concentration of S-nZVI. Despite the changes in soil pH produced by S-nZVI during the treatments, the overall effect of each treatment group on soil pH was relatively limited by day 90. In addition to binding with FeOH, As and Cd can form a complex with FeS on the surface of S-nZVI [33]. Since the doped S in S-nZVI has an extremely strong binding capacity for metal ions, the pH-induced electrostatic adsorption effect on adsorption is not significant. Based on the result of the present experiment, it can be speculated that the synchronous stabilization mechanism of S-nZVI for As and Cd may combine both modes and its immobilization process can be summarized as follows [33]:

(1) $$\equiv \mathrm{FeOH}+\mathrm{HAs}O_{4}^{2-}+{\mathrm{Cd}}^{2+}↔\equiv \mathrm{FeOAs}O_{4}^{2-}{\mathrm{Cd}}^{2+}+{\mathrm{OH}}^{-}$$

(2) $$\equiv \mathrm{FeOH}+{\mathrm{Cd}}^{2+}+H_{2}\mathrm{As}O_{4}^{-}↔\equiv \mathrm{FeOCd}H_{2}\mathrm{As}O_{4}+H^{+}$$

(3) $$\equiv \mathrm{FeOH}+{\mathrm{Cd}}^{2+}+\mathrm{HAs}O_{4}^{2-}↔\equiv \mathrm{FeOCd}H_{2}\mathrm{As}O_{4}$$

(4) $$\equiv \mathrm{FeOH}+\mathrm{Cd}H_{2}\mathrm{As}O_{4}^{+}↔\equiv \mathrm{FeOCd}H_{2}\mathrm{As}O_{4}^{-}+H^{+}$$

(5) ≡FeS+Cd(II)↔≡FeS−Cd(II)

Sulfide modification reduced the agglomeration of particles and increased their surface roughness, thereby enhancing the specific surface area of nZVI [44]. The surface of S-nZVI particles was coated with a thin layer of iron oxide or iron sulfide. For As, the sulfur-doped nature of S-nZVI improves the adsorption and immobilization of As, substantially reducing its environmental mobility and bioavailability through the formation of stable sulfides (e.g., As_2_S_3_) or ferric As compound precipitates [45]. A study quantified the adsorption process of As in aqueous solution by S-nZVI using the Langmuir isothermal model, revealing that the adsorption of As(III) and As(V) on the surface of S-nZVI occurs homogeneously, primarily in monomolecular layers [45]. It was observed that ·OH groups increased with higher S-nZVI dosing, leading to a gradual increase in solution pH, and that oxidation and reduction in As(III) occurred simultaneously within the nanoparticles [32]. Initial rapid immobilization of As can be attributed to the formation and adsorption of As-Fe co-precipitates [46], which is essential for As immobilization, facilitated by the surface corrosion of Fe^0^ [45]. For Cd, the higher specific surface area post-modification may partly explain the highly efficient immobilization of Cd(II) [44]. The reduction in S-nZVI and sulfide production result in the formation of stable cadmium sulfide (CdS), effectively reducing the toxicity and environmental mobility of Cd. Additionally, Cd^2+^ does not undergo reduction during the reaction due to its standard reduction potential being close to that of Fe^2+^ [47]. The synergistic mechanism between FeS and nZVI involves two strategies: sulfide modification inhibits nZVI agglomeration by decreasing its saturation magnetization strength, thus enhancing the specific surface area and reactivity of nZVI; and part of Cd(II) can be removed via complexation with FeS or precipitation with dissolved S^2−^ [44]. Within an appropriate dosing range, the exchangeable content of Cd decreases rapidly, while the proportion of residual or sulfide states increases. However, excessive dosing may alter soil pH or cause excessive iron precipitation, affecting Cd fixation efficiency and potentially leading to secondary pollution.

Overall, there was a significant reduction in As and Cd proportions within the weak acid extraction state in treatment groups supplemented with S-nZVI compared to control groups. This indicates that S-nZVI effectively promotes the transformation of As and Cd in soil into more stable forms while reducing their movement and chemical activity. Furthermore, the remediation using 2 g·kg^−1^ of S-nZVI was more stable and caused less change in soil pH within 90 days. Therefore, the dosage of 2 g·kg^−1^ was used in the subsequent study.

### 3.2. Effect of Soil Moisture Content on Soil As and Cd Fraction

**Figure 4:**
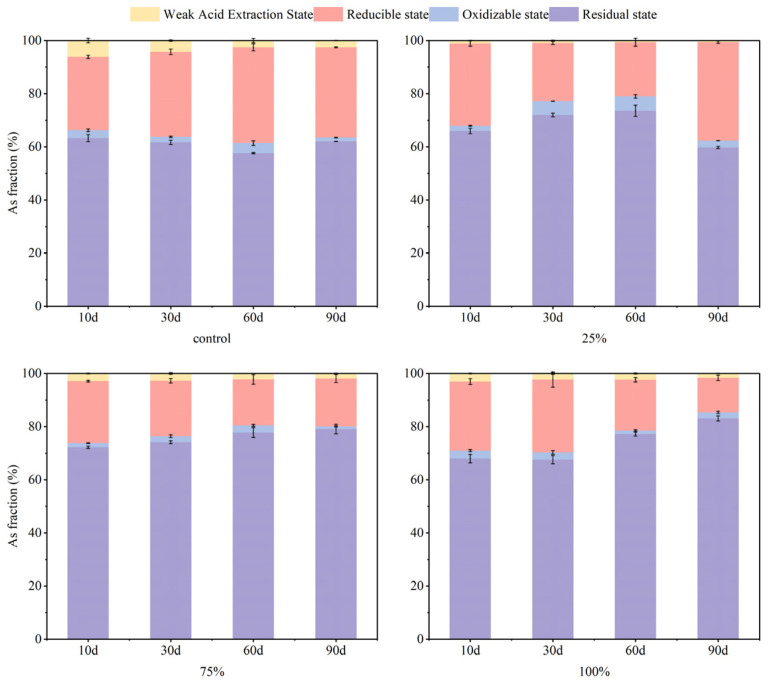
Changes in soil As forms with soil water content before and after addition (total As concentration = 125.32 mg·kg^−1^; water content = 25%, 75%, 100%; S-nZVI concentration = 2 g·kg^−1^).

**Figure 5:**
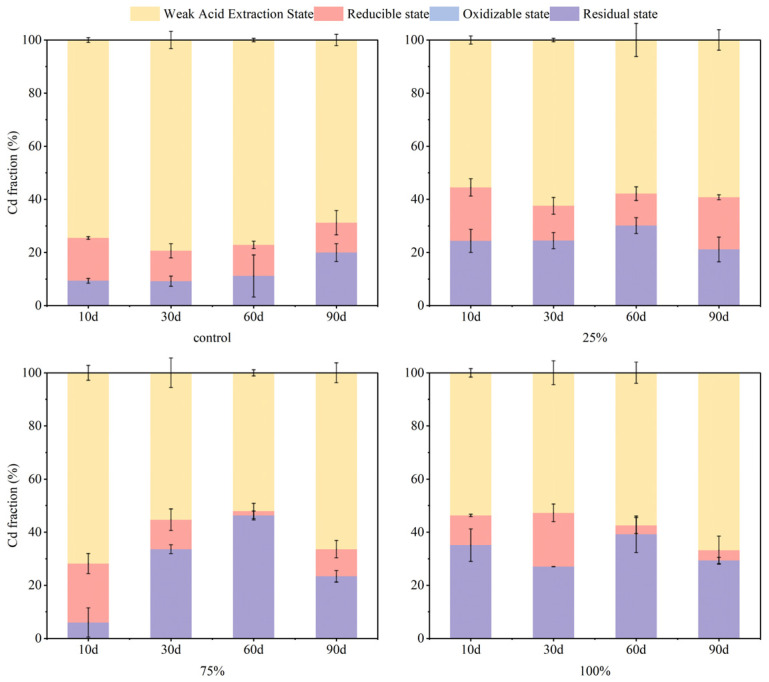
Changes in soil Cd forms with soil water content before and after addition (total Cd concentration = 1.81 mg·kg^−1^; water content = 25%, 75%, 100%; S-nZVI concentration = 2 g·kg^−1^).

Soil moisture conditions affect changes in soil pH, Eh, and sulfur redox state. Eh serves as a critical indicator of the oxidizing or reducing properties of soils, which in turn affects the efficacy of S-nZVI in immobilizing As and Cd, as well as the mechanisms of fraction transformation. At day 60, S-nZVI reduced the mobility of As and Cd across various moisture conditions (25%, 75%, and 100%) compared to control soil. As illustrated in Figure 4, at 25% moisture content, the extension of the experimental period extended to 90 days revealed that the reducible state of As concentrations were higher than that observed in other treatment groups. Additionally, the stability of As in the residual state was reduced, with the possibility of reconverting it to the reduced state, potentially leading to re-reduction from As(V) to As(III), thereby increasing its environmental mobility. This phenomenon may be attributed to the relatively dry state of the soil, which promotes oxidation of the S-nZVI surface and reduces its contact area. In contrast, this phenomenon did not occur under 75% and 100% moisture conditions. The fixation of As by S-nZVI was more stable, with a relatively higher residual state of As at 100% moisture content. This stability is likely due to more consistent and reduced Eh conditions under high moisture, along with the presence of sulfur (-SH) groups or co-precipitates in S-nZVI, which enhance As binding sites [31]. Figure 5 illustrates that, in the short term (10–30 days), S-nZVI reduced the weakly acid-extracted state Cd from 75 to 79% to 55–63% of the control, primarily due to the high reactivity of S-nZVI, which rapidly binds to Cd to form a more stable compound. As the incubation period progressed, the residual state Cd was reduced from 30% to 21% over a period of 60–90 days at 25% soil moisture. At the other two moisture conditions (75% and 100%), the residue state Cd decreased from 39 to 46% to 23–29%, and the corresponding weakly acid extracted state Cd increased from 52 to 57% to 66–67%. This gradual rise in residual-state Cd correlates directly with heightened soil moisture levels. This was attributed to the conversion of soil sulfide to S^2−^ which reacted with Cd^2+^ to form insoluble CdS in the flooded-reducing environment, thus reducing the bioavailability of Cd. Under flooded conditions, the amount of Cd bound to sulfur and ferromanganese complexes increased while the proportion of bioavailable Cd decreased [48].

In general, moisture conditions played a crucial role in modulating the effect of S-nZVI on the immobilization of As and Cd by influencing the redox state of the soil, the activity of S-nZVI and the fraction transformation pathways of the heavy metals. The observed decline in S-nZVI efficacy may stem from its chemical interaction with excess moisture, which formed a thicker passivation layer on the surface, leading to its deterioration and reducing its ability to treat As and Cd.

### 3.3. Effect of pH on Soil As and Cd Fraction

**Figure 6:**
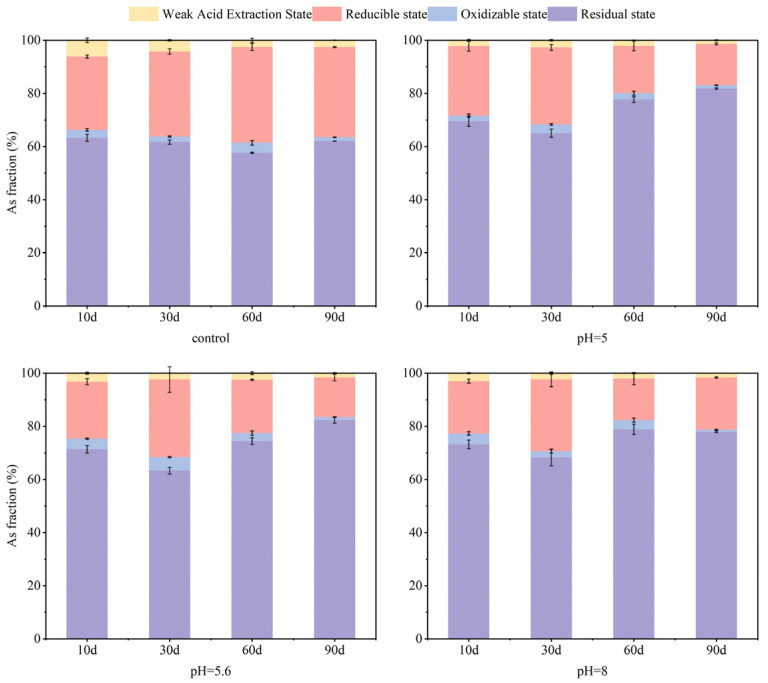
Changes in As forms in soils with different initial pH values (total As concentration = 125.32 mg·kg^−1^, water content = 100%, S-nZVI concentration = 2 g·kg^−1^).

**Figure 7:**
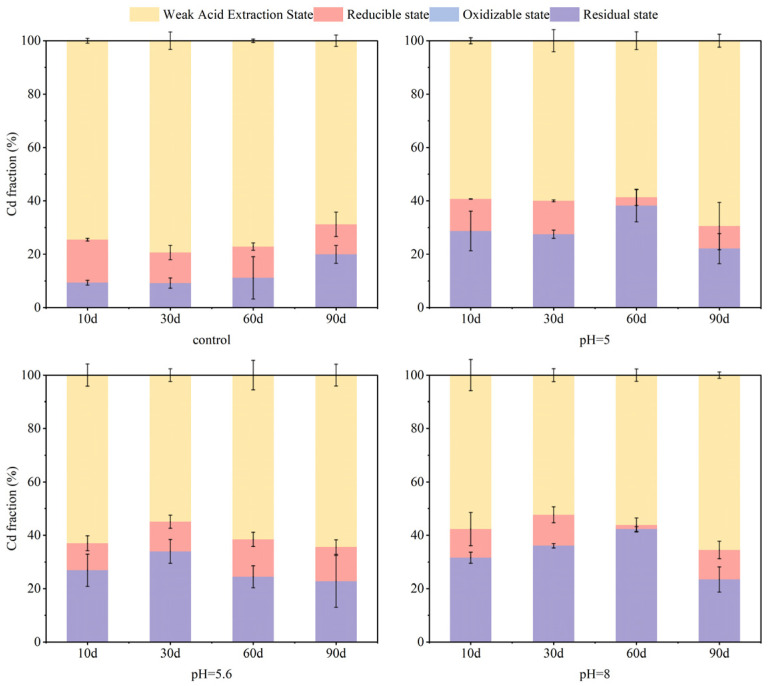
Changes in Cd forms in soils with different initial pH values (total Cd concentration = 1.81 mg·kg^−1^, water content = 100%, S-nZVI concentration = 2 g·kg^−1^).

Soil pH is recognized as a critical parameter reflecting acidity and alkalinity while also serving as an essential determinant influencing heavy metal bioavailability within soils [49]. Furthermore, pH plays an equally crucial role in determining the fraction and activity of As and Cd in soil. After carrying out treatments over 90 days, significant fraction changes for As were observed within the soil, as shown in Figure 6. Residual As levels rose from 62% in untreated soil to between 78 and 82% after treatment, reducing As mobility and bioavailability. The percentage of residual state As (78%) was lower in alkaline soil (initial pH 8.0) than in acidic soil (initial pH 5, 5.6, 82% residual state As), whereas ultimately, reducible state As was slightly higher in alkaline soil (20%) than in acidic soil (15%). These findings align with previous analyses that state that high pH will inhibit As fixation and prompt the conversion of residual state As to reducible state As. This phenomenon may be attributed to the formation of an iron oxide passivation layer via precipitation on the surface of S-nZVI, or alternatively, to a reduction in iron corrosion as pH increases [32]. The surface of S-nZVI generally exhibits a negative charge under alkaline pH conditions, which limits the adsorption of As oxyanions. However, as pH decreases, electrostatic attraction between the S-nZVI surface and As oxyanions enhances immobilization efficiency. At low pH levels, As(V) is more likely to act as a cationic bridge within Fe-As-Cd complexes (Equation (1)). For Cd, comparing remediation effects across 10–60 day time period intervals demonstrates that alkaline environment promotes the fixation of Cd, as shown in Figure 7. On day 60, the residual state of Cd in alkaline soils (42%) surpassed those found in acidic soils (24–38%). By day 90, the corrosive effects on the surface characteristics of S-nZVI, combined with decreasing pH levels, resulted in a portion of the residual Cd being converted to a reducible state. In acidic environments, partial dissolution of S-nZVI leads to decreased Cd(II) immobilization efficiency with decreasing pH, but also releases HS or other sulfur-containing functional groups that react with Cd to form insoluble sulfides [44,50]. At high pH, the negative surface charge enhances electrostatic attraction of Cd ions, facilitating adsorption of Cd(II) onto Fe oxides/hydroxides to form anion-bridged Fe-Cd-As complexes (Equation (2)) [51], thereby promoting Cd immobilization. This further confirms the hypothesis that soil pH affects immobilization: high pH inhibits As immobilization and promotes its conversion from residual to reducible forms, while an alkaline environment enhances Cd immobilization.

Comparing treated and untreated soils, it is evident that initial pH influences As and Cd immobilization by S-nZVI, but due to the strong pH buffering capacity of the soil, the final remediation effect of S-nZVI is less dependent on initial pH. Therefore, in practical applications, S-nZVI can still effectively immobilize As and Cd in soil even if there are fluctuations in soil pH.

## 4. Conclusions

Soil contamination presents critical environmental health and safety challenges, impairing ecosystem services and jeopardizing human health. The presence of heavy metals like As and Cd in soil detrimentally affects agricultural productivity and compromises water quality and biodiversity. Effective remediation of these contaminants is essential for achieving sustainable land use and safeguarding public health. This study evaluates the efficacy of S-nZVI in remediating As and Cd in contaminated soils, providing valuable insights into effective strategies for mitigating soil pollution. The factors examined include the dosage of S-nZVI, soil moisture conditions, and initial soil pH values. It was found that S-nZVI efficiently converted As and Cd in soil from unstable reducible states and weak acid extraction states to stable residual states. The following conclusions can be drawn:

(1) S-nZVI dosage exhibited a pronounced dose-dependent effect on both remediation efficiency and long-term stability of As and Cd. Increasing the dosage resulted in a reduction in As content within the weak acid extraction state. During the 90-day experimental time, the concentration of 5 g·kg^−1^ S-nZVI effectively maintained As content within 1% in this state while promoting the conversion of most reduced state As into more stable residual forms. For Cd, after 60 days, residual state Cd concentrations reached 36%, 39%, and 46% for treatment groups receiving dosages of 1 g·kg^−1^, 2 g·kg^−1^, and 5 g·kg^−1^ respectively, indicating that higher dosages enhanced immobilization effects on Cd. By day 90, although some residual state Cd had transformed back to weak acid extraction states, the higher the dosage and the more obvious transformation, the higher the proportion of residual state Cd. The dosage of 2 g·kg^−1^ yielded optimal remediation outcomes for both As and Cd.
(2) Soil moisture conditions influenced reduced forms of As and Cd present in soils. Within the first 60 days, S-nZVI markedly decreased mobility for both contaminants. The fixation rates for As remained relatively stable throughout this period. With the extension of the experimental period up to 90 days, the content of residual state As was 83% at 100% water content. For Cd, in the short-term incubation (10–30 days), S-nZVI reduced the weakly acid-extracted state of Cd as the incubation time progressed. Meanwhile, in the period of 60–90 days, an increase was noted alongside a decrease in residual state Cd content. The highest content of residual state Cd was observed at a water content of 100%. The decay of the performance of S-nZVI could be attributed to its chemical interaction with excess water, which led to its aging and reduced capacity for As and Cd.
(3) The application of S-nZVI across different initial pH values (5, 5.6, and 8, respectively) led to significant increases in residual As content, from 62% in untreated soils to 78–82%. After S-nZVI treatment, the content of Cd in the weakly acidic extractive state of the soil was maintained at 64–69% in all cases. Given the strong buffering capacity of soil against pH fluctuations, the final remediation effect of S-nZVI had a limited dependence on the initial pH of the soil.

In summary, S-nZVI demonstrated substantial effectiveness in reducing the bioavailability of As and Cd in the soil, specifically under conditions of 100% moisture content and dosage at 2 g·kg^−1^ within 90 days. The findings provide critical insights for designing field-scale remediation strategies for heavy metal co-contaminated paddy soils.

## Figures and Tables

**Table 1:** Basic physical and chemical properties of soil.

pH	Organic Matter (g·kg^−1^)	Total Concentration (mg·kg^−1^)	
		As	Cd
5.6	113.65	125.32	1.81

## Data Availability

The original contributions presented in this study are included in the article. Further inquiries can be directed to the corresponding authors.
